# Supplementary material for: Impact of COVID-19 Restriction on Weight, Physical Activity, Diet and Psychological Distress on People with PCOS
Source: Nutrients. 2023 May 31;15(11):2579. doi: 10.3390/nu15112579 (PMC10255147; doi:10.3390/nu15112579)
Supplement: Supplementary file 1 [file nutrients-15-02579-s001.zip › nutrients-2381016-supplementary.pdf]

Supplementary Table S1. Chronological order of COVID-19 restrictions enforced throughout 2020 by state.

Date	Announcement	States restrictions were implemented
23 March 2020	Restrictions implemented<br>• Temporary closure of non-essential activities and businesses	ACT, NSW, QLD, SA, VIC, WA
23 March 2020	Restrictions implemented<br>• Closer of gyms and bars, and to limit the number of attendees at weddings and funerals.<br>• Remote communities were exempt from these restrictions<br>• Children school attendance options for remainder of term	NT
27 March 2020	Restrictions implemented<br>• Everyone required to be in self-quarantine	NT
30 March 2020	Restrictions implemented<br>• Stay at home order (unless attending work or school, getting essential or medical supplies, exercising or providing compassionate care)	TAS
30 March 2020	Restrictions implemented<br>• Stay at home order for non-essential workers (unless getting essential or medical supplies, exercising or providing compassionate care)<br>• Regional boundaries established (no travel between regions)	WA
30 March 2020	Restrictions implemented<br>• Stay at home order (unless attending work or school, getting essential or medical supplies, exercising or providing compassionate care)<br>• Gatherings limited to two people	VIC, NSW, ACT, QLD, SA
12 April 2020	Restrictions implemented (Northwest Tasmanian)<br>• Schools, hospitals and businesses closed.	TAS
1 May 2020	Restrictions eased<br>• Two visitors allowed at homes<br>• Allowed to leave for non-essential shopping	ACT
1 May 2020	Restrictions eased<br>• Recommencement of outdoor sports (non-contact)<br>• Opening of public pools	NT
4 May 2020	Restriction eased (Northwest Tasmanian)<br>• Schools, hospitals and essential businesses reopened	TAS

11 May 2020	Easing restriction and roadmap announced	SA
11 May 2020	Restrictions eased<br>• 20 people to attend funerals<br>• Visitors allowed at residential aged care once a week<br>• TasTAFE campuses and training facilities to open for small groups of students attending practical learning and assessments	TAS
11 May 2020	Restriction eased<br>• Maximum gatherings ten people outdoors<br>• Maximum five people visiting at someone's home<br>• Maximum 10 guests at weddings<br>• Maximum 20 people at funeral indoors and 30 people outdoors<br>• Resumption of some outdoor recreational activities	VIC
15 May 2020	Restriction eased<br>• Cafés and restaurants to seat up to ten people at one time	ACT, NSW
15 May 2020	Stage two activities would commence from 15 May 2020	NT
15 May 2020	Restrictions eased<br>• Maximum gathering ten people in a public space<br>• Recreational travel of maximum 150km from home for day trips<br>• Re-opening of libraries, playground equipment, skate parks and outdoor gyms, with a maximum of ten people at one time<br>Stage two of easing restrictions would be from 12 June 2020	QLD
18 May 2020	Restrictions eased<br>• Indoor and outdoor non-work gatherings lifted to 20 people<br>• Return to work, unless they are unwell or vulnerable<br>• Cafés and restaurants can reopen with meal service (including within pubs, bars, clubs, hotels and casino), maximum 20 patrons and the four-square metre rule applied<br>• Weddings and funerals maximum 20 attendees (30 for outdoor)<br>• Places of worship, community facilities and libraries to re-open, limited to 20 patrons<br>• Community sports (non-contact) limited to 20 people<br>• Outdoor or indoor fitness classes limited to 20 participants<br>• Public swimming pools can open, limited to 20 patrons per pool.	WA

30 June 2020	Restrictions implemented<br>• Stay at home order for metropolitan Melbourne (3038, 3064, 3047, 3060, 3012, 3032, 3055, 3042, 3021, 3046)	VIC
8 July 2020	Restrictions implemented<br>• Stay at home orders for Melbourne (3000) and Mitchell Shire (3163)	VIC
9 July 2020	Restrictions implemented<br>• Stay at home orders for Flemmington (3031) and North Melbourne (3051)	VIC
31 July 2020	Restrictions implemented<br>• Stay at home order for Colac-Otway, Greater Geelong, Surf Coast, Moorabool, Golden Plains, and the Borough of Queenscliffe	VIC
2 August 2020	Restrictions implemented<br>• Stay at home order for entire metropolitan Melbourne area and restrictions increased	VIC
3 August 2020	Restrictions implemented<br>• Stay at home order for all of regional Victoria	VIC
16 September 2020	Restriction eased (regional Victoria)<br>• Stay at home order lifted<br>• Visitors allowed at homes	VIC
18 October 2020	Restrictions eased (metropolitan Melbourne)<br>• Stay at home order lifted<br>• No visitors at homes allowed<br>• Gathering outside allowed	VIC

ACT, Australian Capital Territory; NT, Northern Territory; NSW, New South Wales; QLD, Queensland; SA, South Australia; TAS, Tasmania; VIC, Victoria; WA, Western Australia. Table outlines restrictions implemented in response to COVID-19 transmission, each state was responsible for their own restrictions. (1) (2).

1. Australia Po. COVID-19: a chronology of Australian Government announcements (up until 30 June 2020) Canberra, ACT: Australian Government 2021 [Available from: https://www.aph.gov.au/About_Parliament/Parliamentary_departments/Parliamentary_Library/pubs/rp/rp2021/Chronologies/COVID-19AustralianGovernmentAnnouncements.
2. Andrews D. News and updates from Dan Andrews and his team. VIC, Australia: Premier of Victoria 2020 [Available from: https://www.premier.vic.gov.au/?filters%5Bfield_news_date%5D%5Btype%5D=date&filters%5Bfield_news_date%5D%5Boperator%5D=lte&filters%5Bfield_news_date%5D%5Bvalues%5D=2020-06-30T00%3A00%3A00%2B10%3A00.

Supplementary Table S2. Multivariable Linear and Logistic Regression for Weight Changes

		Weight Change (kg)		Weight Change (%)		Binary Weight change	
		Beta (95% CI)	P Value	Beta (95% CI)	P Value	OR (95% CI)	P Value
PCOS	None	Ref	Ref	Ref	Ref	Ref	Ref
	Diagnosis	-0.09 (-1.63, 1.45)	0.911	1.61 (0.027, 3.20)	0.046	1.09 (0.701, 1.71)	0.694
Location	Metropolitan Melbourne	Ref	Ref	Ref	Ref	Ref	Ref
	Other	0.94 (-0.246, 2.13)	0.120	0.008 (-1.22, 1.24)	0.280	1.29 (0.915, 1.81)	0.146
Age (years)	18-24	Ref	Ref	Ref	Ref	Ref	Ref
	25-34	0.08 (-1.57, 1.74)	0.921	-0.22 (-1.93, 1.49)	0.802	0.86 (0.520, 1.41)	0.541
	35-44	0.60 (-1.17, 2.37)	0.506	-0.33 (-2.16, 1.49)	0.720	1.28 (0.762, 2.15)	0.351
	45+	-0.24 (-2.12, 1.64)	0.800	-0.77 (-2.71, 1.16)	0.434	1.00 (0.567, 1.750)	0.990
Education	University	Ref	Ref	Ref	Ref	Ref	Ref
	Tafe/certificate	1.66 (0.114, 3.21)	0.035	-0.35 (-1.96, 1.25)	0.666	0.63 (0.398, 0.983)	0.042
	Secondary	0.82 (-0.282, 2.22)	0.252	-0.89 (-2.34, 0.56)	0.229	0.68 (0.452, 1.025)	0.065
Employment	Student	Ref	Ref	Ref	Ref	Ref	Ref
	Part time	0.65 (-1.79, 3.09)	0.601	3.56 (1.03, 6.08)	0.006	1.66 (0.77, 3.56)	0.195
	Full time	0.89 (-1.56, 3.34)	0.476	4.24 (1.71, 6.77)	0.001	2.06 (0.950, 4.48)	0.067
	Other	1.12 (-1.42, 3.67)	0.386	3.92 (1.27, 6.57)	0.004	1.76 (0.0801, 3.88)	0.159
Income (AUD$)	0-24,999	Ref	Ref	Ref	Ref	Ref	Ref
	25,000-49,999	-2.03 (-4.59, 0.527)	0.119	-1.38 (-4.01, 1.25)	0.303	1.01 (0.517, 2.37)	0.794
	50,000-124,999	-2.28 (-4.47, -0.097)	0.041	-2.20 (-4.43, 0.04)	0.054	0.74 (0.382, 1.44)	0.374

	125,000-149,999	-0.86 (-3.42, 1.69)	0.507	-0.45 (-3.06, 2.17)	0.737	1.43 (0.662, 3.07)	0.364
	150,000+	-1.68 (-4.05, 0.701)	0.167	-1.60 (-4.04, 0.83)	0.197	1.00 (0.485, 2.05)	0.993
Food security	High	Ref	Ref	Ref	Ref	Ref	Ref
	Marginal	-0.26 (-1.77, 1.25)	0.734	-0.93 (-2.49, 0.63)	0.242	0.86 (0.544, 1.35)	0.510
	Low	0.49 (-1.04, 2.01)	0.532	0.78 (-0.78, 2.34)	0.326	1.28 (0.818, 2.01)	0.279
	Very low	-0.17 (-2.07, 1.74)	0.864	-0.43 (-2.39, 1.52)	0.663	1.03 (0.581, 1.82)	0.923
Culture	Oceania	Ref	Ref	Ref	Ref	Ref	Ref
	European	-0.45 (-1.65, 0.749)	0.460	0.15 (-1.11, 1.40)	0.818	0.93 (0.652, 1.33)	0.698
	Asian	-0.13 (-1.68, 1.42)	0.868	0.93 (-0.65, 2.50)	0.246	1.25 (0.787, 1.99)	0.341
	Other	-2.22 (-5.42, 0.983)	0.174	-2.55 (-6.00, 0.90)	0.147	0.64 (0.249, 1.63)	0.348
Number of children	0	Ref	Ref	Ref	Ref	Ref	Ref
	1	0.35 (-1.10, 1.81)	0.634	0.56 (-0.95, 2.07)	0.469	0.91 (0.598, 1.39)	0.670
	2	0.69 (-0.698, 2.08)	0.329	0.64 (-0.79, 2.07)	0.377	1.19 (0.789, 1.787)	0.411
	3+	0.29 (-1.58, 2.16)	0.761	0.74 (-2.74, 3.10)	0.445	0.98 (0.559, 1.70)	0.929

Footnotes: Data is presented as beta (95% confidence interval) for continuous and odds ratio (95% confidence interval) for categorical data and were analysed with linear and logistic regression, respectively. Ref: reference value; AUD$: Australian dollars; PCOS: polycystic ovary syndrome

Supplementary Table S3. Multivariable Linear and Logistic Regression for Psychological Distress and Physical Activity.

		PA Categories		K10 Continuous Score		K10 Categories	
		OR 95% CI	P Value	Beta 95% CI	P Value	OR 95% CI	P Value
PCOS	None	Ref	Ref	Ref	Ref	Ref	Ref
	Diagnosis	0.50 (0.32, 0.79)	0.003	0.20 (-1.65, 2.04)	0.834	1.15 (0.72, 1.84)	0.563
Location	Metropolitan Melbourne	Ref	Ref	Ref	Ref	Ref	Ref
	Other	1.33 (0.93, 1.90)	0.115	2.02 (0.62, 3.42)	0.005	1.61 (1.13, 2.29)	0.008
Age (years)	18-24	Ref	Ref	Ref	Ref	Ref	Ref
	25-34	0.48 (0.28, 0.84)	0.010	-2.15 (-4.17, -0.14)	0.036	0.57 (0.35, 0.95)	0.031
	35-44	0.38 (0.21, 0.67)	0.001	-4.30 (-6.43, -2.18)	0.000	0.34 (0.20, 0.59)	0.000
	45+	0.41 (0.22, 0.75)	0.004	-4.74 (-7.04, -2.44)	0.000	0.37 (0.21, 0.67)	0.001
Education	University	Ref	Ref	Ref	Ref	Ref	Ref
	Tafe/certificate	1.73 (1.09, 2.74)	0.020	-1.56 (-3.42, 0.30)	0.099	0.71 (0.45, 1.13)	0.150
	Secondary	2.19 (1.43, 3.3)	0.000	-1.36 (-3.03, 0.32)	0.112	0.83 (0.55, 1.27)	0.396
Employment	Student	Ref	Ref	Ref	Ref	Ref	Ref
	Part time	0.48 (0.22, 1.06)	0.070	2.73 (-0.20, 5.65)	0.068	1.21 (0.58, 2.53)	0.613
	Full time	0.51 (0.23, 1.14)	0.101	2.74 (-0.23, 5.70)	0.071	1.25 (0.59, 2.65)	0.554
	Other	0.43 (0.19, 0.96)	0.041	3.06 (0.05, 6.07)	0.047	1.26 (0.59, 2.71)	0.549
Income (AUD$)	0-24,999	Ref	Ref	Ref	Ref	Ref	Ref
	25,000-49,999	0.66 (0.31, 1.43)	0.294	-4.49 (-7.59, -1.40)	0.005	0.38 (0.17, 0.85)	0.018
	50,000-124,999	1.66 (0.85, 3.24)	0.141	-4.25 (-6.97, -1.53)	0.002	0.39 (0.19, 0.79)	0.009
	125,000-149,999	2.30 (1.04, 5.09)	0.040	-6.17 (-9.35, -2.98)	0.000	0.28 (0.12, 0.64)	0.002
	150,000+	2.36 (1.12, 4.94)	0.023	-7.36 (-10.33, -4.40)	0.000	0.16 (0.07, 0.35)	0.000
Food security	High	Ref	Ref	Ref	Ref	Ref	Ref
	Marginal	0.87 (0.55, 1.39)	0.573	-1.15 (-2.98, 0.68)	0.216	0.68 (0.42, 1.11)	0.122
	Low	0.80 (0.51, 1.27)	0.349	-1.82 (-3.67, 0.02)	0.052	0.64 (0.40, 1.04)	0.071
	Very low	1.37 (0.75, 2.52)	0.307	-0.09 (-2.40, 2.21)	0.936	1.03 (0.58, 1.83)	0.910
Culture	Oceania	Ref	Ref	Ref	Ref	Ref	Ref
	European	0.98 (0.68, 1.42)	0.915	0.88 (-0.56, 2.33)	0.231	1.29 (0.90, 1.87)	0.167
	Asian	0.56 (0.35, 0.90)	0.016	-1.40 (-3.31, 0.52)	0.152	0.67 (0.40, 1.10)	0.114

	Other	0.80 (0.32, 2.01)	0.630	-1.49 (-5.12, 2.14)	0.421	0.69 (0.26, 1.81)	0.449
Number of children	0	Ref	Ref	Ref	Ref	Ref	Ref
	1	0.68 (0.45, 1.05)	0.081	-1.37 (-3.07, 0.34)	0.117	0.82 (0.53, 1.26)	0.359
	2	0.84 (0.55, 1.27)	0.403	-2.00 (-3.67, -0.32)	0.019	0.62 (0.39, 0.96)	0.033
	3+	1.04 (0.59, 1.83)	0.885	-0.83 (-3.07, 1.41)	0.466	0.68 (0.37, 1.22)	0.196

Footnotes: Data is presented as beta (95% confidence interval) for continuous and odds ratio (95% confidence interval) for categorical data and were analysed with linear and logistic regression, respectively. Ref: reference value; AUD$: Australian dollars; PCOS: polycystic ovary syndrome

Supplementary Table S4. Multivariable Linear and Logistic Regression for Dietary Intake.

		Fruit		Vegetable		Sugar Sweetened Beverage		Discretionary Foods		Alcohol	
		Beta (95% CI)	P Value	Beta (95% CI)	P Value	OR (95% CI)	P Value	OR (95% CI)	P Value	OR (95% CI)	P Value
PCOS	None	Ref	Ref	Ref	Ref	Ref	Ref	Ref	Ref	Ref	Ref
	Diagnosis	0.03 (-0.21, 0.27)	0.778	0.23 (-0.11, 0.56)	0.179	1.74 (1.10, 2.75)	0.019	0.65 (0.32, 1.32)	0.235	0.70 (0.45, 1.10)	0.125
Location	Metropolitan Melbourne	Ref	Ref	Ref	Ref	Ref	Ref	Ref	Ref	Ref	Ref
	Other	-0.06 (-0.24, 0.13)	0.547	-0.05 (-0.30, 0.20)	0.703	0.95 (0.67, 1.34)	0.767	0.81 (0.49, 1.33)	0.405	0.80 (0.57, 1.11)	0.184
Age (years)	18-24	Ref	Ref	Ref	Ref	Ref	Ref	Ref	Ref	Ref	Ref
	25-34	0.12 (-0.14, 0.39)	0.352	-0.18 (-0.55, 0.18)	0.324	1.26 (0.77, 2.08)	0.357	0.89 (0.47, 1.70)	0.726	0.99 (0.61, 1.62)	0.982
	35-44	0.11 (-0.16, 0.38)	0.419	-0.07 (-0.45, 0.32)	0.732	1.50 (0.89, 2.53)	0.131	0.48 (0.24, 0.98)	0.045	1.12 (0.67, 1.87)	0.664
	45+	0.09 (-0.21, 0.39)	0.560	-0.05 (-0.47, 0.37)	0.798	1.03 (0.58, 1.81)	0.932	0.81 (0.39, 1.68)	0.566	1.29 (0.74, 2.26)	0.372
Education	University	Ref	Ref	Ref	Ref	Ref	Ref	Ref	Ref	Ref	Ref
	Tafe/certificate	0.02 (-0.22, 0.26)	0.854	-0.13 (-0.46, 0.20)	0.439	0.84 (0.54, 1.32)	0.459	1.96 (1.09, 3.53)	0.026	1.22 (0.78, 1.92)	0.383
	Secondary	-0.05 (-0.27, 0.17)	0.632	-0.10 (-0.40, 0.20)	0.514	0.71 (0.47, 1.08)	0.107	0.83 (0.46, 1.49)	0.529	0.97 (0.65, 1.47)	0.903
Employment	Student	Ref	Ref	Ref	Ref	Ref	Ref	Ref	Ref	Ref	Ref
	Part time	-0.02 (-0.40, 0.36)	0.922	0.22 (-0.30, 0.75)	0.405	0.68 (0.33, 1.40)	0.298	2.02 (0.69, 5.90)	0.197	1.46 (0.71, 2.98)	0.302
	Full time	-0.20 (-0.58, 0.18)	0.302	0.31(-0.22, 0.84)	0.255	0.58 (0.28, 1.19)	0.136	2.15 (0.73, 6.32)	0.166	1.07 (0.52, 2.21)	0.853
	Other	-0.13 (-0.52, 0.26)	0.514	0.47 (-0.07, 1.00)	0.089	0.58 (0.28, 1.20)	0.141	1.90 (0.64, 5.69)	0.250	0.96 (0.46, 2.01)	0.910
Income (AUD$)	0-24,999	Ref	Ref	Ref	Ref	Ref	Ref	Ref	Ref	Ref	Ref
	25,000-49,999	0.06 (-0.34, 0.47)	0.763	-0.60 (-1.1, -0.04)	0.036	0.91 (0.43, 1.92)	0.807	0.94 (0.34, 2.60)	0.906	0.84 (0.40, 1.76)	0.639

	50,000-124,999	0.07 (-0.29, 0.43)	0.711	-0.53 (-1.03, -0.035)	0.036	1.00 (0.52, 1.93)	0.993	1.12 (0.47, 2.69)	0.801	1.04 (0.54, 1.98)	0.907
	125,000-149,999	0.11 (-0.31, 0.53)	0.610	-0.19 (-0.76, 0.39)	0.521	0.66 (0.30, 1.45)	0.302	0.91 (0.32, 2.57)	0.860	1.05 (0.49, 2.25)	0.891
	150,000+	0.12 (-0.27, 0.51)	0.555	-0.67 (-1.21, -0.12)	0.016	1.40 (0.69, 2.87)	0.353	0.74 (0.28, 2.00)	0.554	0.90 (0.44, 1.81)	0.759
Food security	High	Ref	Ref	Ref	Ref	Ref	Ref	Ref	Ref	Ref	Ref
	Marginal	-0.16 (-0.40, 0.08)	0.191	-0.04 (-0.37, 0.30)	0.829	1.38 (0.88, 2.16)	0.167	1.51 (0.83, 2.72)	0.175	0.99 (0.64, 1.54)	0.972
	Low	0.10 (-0.13, 0.34)	0.395	0.11 (-0.22, 0.43)	0.516	1.35 (0.87, 2.11)	0.183	1.20 (0.64, 2.26)	0.560	1.02 (0.66, 1.59)	0.923
	Very low	0.17 (-0.13, 0.46)	0.276	0.22 (-0.19, 0.62)	0.291	0.92 (0.52, 1.64)	0.784	1.50 (0.74, 3.06)	0.260	0.77 (0.43, 1.35)	0.357
Culture	Oceania	Ref	Ref	Ref	Ref	Ref	Ref	Ref	Ref	Ref	Ref
	European	-0.14 (-0.33, 0.04)	0.127	-0.14 (-0.40, 0.13)	0.307	0.62 (0.43, 0.89)	0.010	1.23 (0.75, 2.00)	0.411	0.80 (0.56, 1.14)	0.215
	Asian	0.02 (-0.22, 0.26)	0.892	0.04 (-0.29, 0.38)	0.803	1.10 (0.69, 1.75)	0.695	1.19 (0.61, 2.31)	0.610	1.02 (0.64, 1.62)	0.946
	Other	-0.11 (-0.57, 0.35)	0.643	0.65 (0.04, 1.27)	0.038	1.39 (0.56, 3.45)	0.484	1.12 (0.315, 4.00)	0.859	1.39 (0.58, 3.37)	0.461
Number of children	0	Ref	Ref	Ref	Ref	Ref	Ref	Ref	Ref	Ref	Ref
	1	0.12 (-0.14, 0.39)	0.352	-0.09 (-0.39, 0.20)	0.539	0.78 (0.51, 1.18)	0.237	0.89 (0.47, 1.70)	0.726	0.99 (0.61, 1.62)	0.982
	2	0.11 (-0.16, 0.38)	0.419	0.09 (-0.21, 0.39)	0.571	0.87 (0.58, 1.31)	0.506	0.48 (0.24, 0.99)	0.045	1.12 (0.67, 1.87)	0.664
	3+	0.09 (-0.21, 0.39)	0.560	0.45 (0.04, 0.85)	0.032	0.71 (0.41, 1.25)	0.237	0.81 (0.39, 1.68)	0.566	1.29 (0.74, 2.26)	0.372

Footnotes: Data is presented as beta (95% confidence interval) for continuous and odds ratio (95% confidence interval) for categorical data and were analysed with linear and logistic regression, respectively. Ref: reference value; AUD$: Australian dollars; PCOS: polycystic ovary syndrome
